# Theoretical study of D–A′–π–A/D–π–A′–π–A triphenylamine and quinoline derivatives as sensitizers for dye-sensitized solar cells†

**DOI:** 10.1039/d0ra01040e

**Published:** 2020-05-04

**Authors:** Ying Zhang, Ji Cheng, Wang Deng, Bin Sun, Zhixin Liu, Lei Yan, Xueye Wang, Baomin Xu, Xingzhu Wang

**Affiliations:** College of Chemistry, School of Physics and Optoelectronics, Xiangtan Univeristy Xiangtan Hunan 411105 China xz_wang@hotmail.com yanlei@xtu.edu.cn wxueye@xtu.edu.cn; Department of Materials Science & Engineering, Academy for Advanced Interdisciplinary Research, Southern University of Science and Technology Shenzhen Guangdong 518055 China xubm@sustech.edu.cn; North China Sea Marine Forecasting Center of Ministry of Natural Resources Qingdao Shandong 266000 China

## Abstract

We have designed four dyes based on D–A′–π–A/D–π–A′–π–A triphenylamine and quinoline derivatives for dye-sensitized solar cells (DSSCs) and studied their optoelectronic properties as well as the effects of the introduction of alkoxy groups and thiophene group on these properties. The geometries, single point energy, charge population, electrostatic potential (ESP) distribution, dipole moments, frontier molecular orbitals (FMOs) and HOMO–LUMO energy gaps of the dyes were discussed to study the electronic properties of dyes based on density functional theory (DFT). And the absorption spectra, light harvesting efficiency (LHE), hole–electron distribution, charge transfer amount from HOMO to LUMO (*Q*_CT_), *D* index, *H*_CT_ index, *S*_m_ index and exciton binding energy (*E*_coul_) were discussed to investigate the optical and charge-transfer properties of dyes by time-dependent density functional theory (TD-DFT). The calculated results show that all the dyes follow the energy level matching principle and have broadened absorption bands at visible region. Besides, the introduction of alkoxy groups into triarylamine donors and thiophene groups into conjugated bridges can obviously improve the stability and optoelectronic properties of dyes. It is shown that the dye D4, which has had alkoxy groups as well as thiophene groups introduced and possesses a D–π–A′–π–A configuration, has the optimal optoelectronic properties and can be used as an ideal dye sensitizer.

## Abstract 2

We have designed four dyes based on D–A′–π–A/D–π–A′–π–A triphenylamine and quinoline derivatives for DSSCs and studied their optoelectronic properties as well as the effects of the introduction of alkoxy groups and thiophene group on the properties.

## Graphical abstract

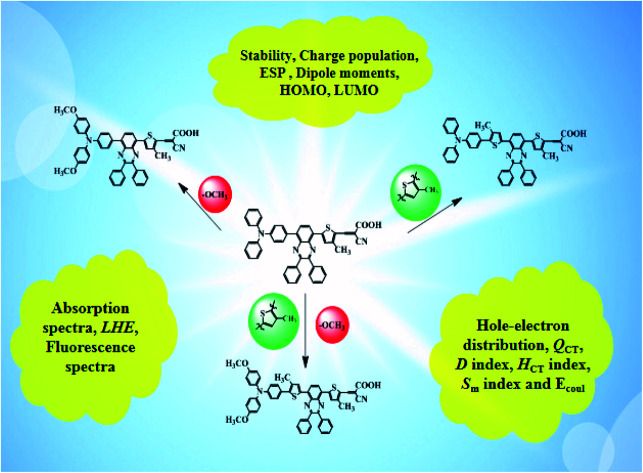

## 1. Introduction

Since O'Regan and Grätzel^1^ first proposed dye-sensitized solar cells in 1991, dye-sensitized solar cells (DSSCs) have begun to attract more and more attention from researchers around the world.^2–4^ Compared with traditional silicon cells, DSSCs are easier to synthesize,^5^ environmentally friendly,^6^ cost-effective,^7^ and can display highly impressive photoelectric conversion efficiency 14.3% (ref. 8) under standard illumination. DSSCs, among which sensitizers play a crucial role in improving energy conversion efficiency, are mainly composed of a mesoporous semiconductor film, sensitizer and redox electrolytes.^5–8^ The ideal dye sensitizers need to satisfy the following:^5–12^ (a) narrow band gap, the LUMO level of the dye is above the semiconductor conduction band, to provide stable charge transfer channel and fast electron injection; (b) there are wide and strong absorption bands in the visible and near-infrared regions to ensure effective charge separation and improve the overall solar energy conversion efficiency; (c) sufficient photothermal stability to make sure that DSSCs devices can work for a long time. Therefore, these requirements provide the direction for us to design better dyes. As is known to us, donor–π–acceptor (D–π–A) system is the basic structure of the molecular dyes in nonmetallic dye-sensitized solar cells.^9–11^ For the past few years, the additional electron-withdrawing group has been introduced into the D–π–A system to adjust the energy gap of dyes, thus a new D–A′–π–A system was developed. The addition of the auxiliary acceptor group will be helpful to extend the effective length of the conjugated structure, broaden the absorption region, so as to improve the spectral response and light-heat stability of the photosensitizer.^12–14^ At the same time, there have been reports on D–π–A′–π–A system with the introduction of an additional electron-donating group into the D–A′–π–A system. The D–π–A′–π–A system^15–17^ has been proved to effectively improve the flatness of the donor to the auxiliary acceptor, which is favorable for better charge separation and smoother intramolecular charge transfer (ICT) in photoexcited process. What's more, a better plane structure is obviously conducive to improve the ability of light harvesting, avoid energy loss from reverse relaxation, so as to improve the overall photoelectric conversion efficiency (PCE) of the solar cells. The triarylamine, with excellent electron-donating ability, is commonly used as electron donor.^18–20^ The cyanoacrylic acid, with prominent electron-withdrawing ability, is generally used as electron acceptor and anchor group absorbed on the surface of the semiconductor.^21,22^ The quinoline,^23,24^ with good electron-withdrawing ability, is often used as auxiliary acceptor. Thiophene and its derivatives,^25,26^ with high polarizability, are usually used as conjugated bridge.

**Fig. 1:**
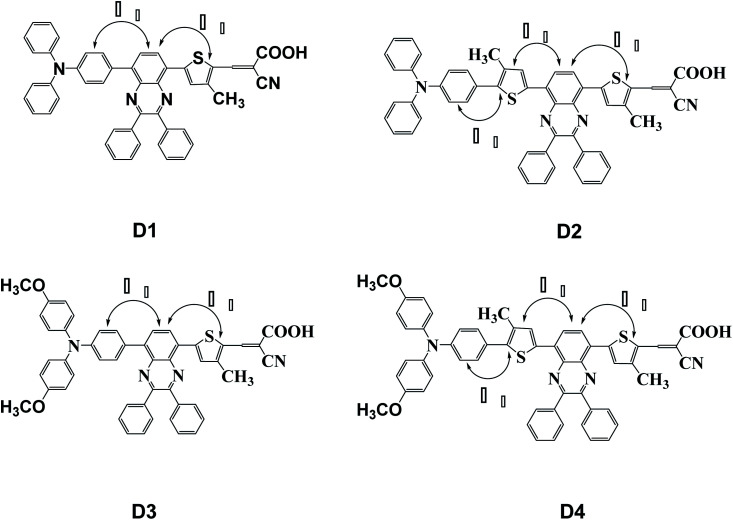
Molecular structures of dyes D1–D4.

In this paper, four dye molecules D1–D4 (as shown in Fig. 1), based on triphenylamine (TPA) and quinoline derivatives, have been designed. The triarylamine was applied as electron donor, cyanoacrylic acid as electron acceptor, quinoline as auxiliary electron acceptor, and thiophene group as π bridge. D1 and D3 are both typical D–π–A′–π–A configurations, D2 and D4 are both typical D–π–A′–π–A configurations. Compared with D2 and D1 respectively, D4 and D3 are respectively introduced in oxyalkyl chains. And compared with D3 and D1 respectively, D4 and D2 are respectively introduced in a thiophene group. The geometrical structure, electronic properties, optical properties and intramolecular charge transfer properties of the four dyes were studied based on density functional theory (DFT) and time-dependent density functional theory (TD-DFT).

## 2. Computational details

The geometrical structures of the dyes were optimized by using B3LYP functional method based on density functional theory (DFT) at 6-311g(d,p) basis set level in vacuum.^27–30^ Because all molecules are asymmetric, no symmetry restriction was applied. At the same theoretical level, the calculated frequency results show that all the optimized molecular structures have no imaginary frequency, indicating that the obtained geometrical configurations are stable. Based on the optimized structure, the dipole moments, the highest occupied molecular orbitals (HOMOs), the lowest unoccupied molecular orbitals (LUMOs) and band gaps of the dyes were calculated. The surface electrostatic potential (ESP) distribution diagram of the dyes were simulated with an electron density of 0.0004 a.u. The single point energy of the dyes was obtained by DFT//B3LYP/def2TZVP.^31–34^ The charge population of the dyes were acquired through the natural bond orbital (NBO) calculation.^35^ The Gaussian 09 software package^36^ was used for all DFT calculations. Charge population analysis was performed using Multiwfn 3.6(dev)^37^ program.

An ideal dye sensitizer requires a wide and strong absorption band in the visible and near infrared region to ensure effective charge separation and improve the overall solar energy conversion efficiency. The short-circuit current density (*J*_SC_) of dye-sensitized solar cells is positively correlated with the light harvesting efficiency LHE(*λ*) of dye molecules. The value of LHE(*λ*) can be given through the following formula:^24,26,38–40^

1 LHE(*λ*) = 1 − 10^−*f*^

*f* represents the oscillator strength at a certain wavelength. If the value of *f* is larger, the value of LHE(*λ*) and *J*_SC_ will be both larger. Based on the optimized structures, the corresponding minimum 30 single transition electronic excited states were studied based on the time-dependent density functional theory (TD-DFT) with BHandHLYP^39,40^ functional at 6-311g(d,p) basis set level. The ultraviolet-visible (UV-VIS) spectra of the dyes were simulated ground on Gaussian function and the calculated transition data. The default half-peak width (0.333 eV) was adopted for the ultraviolet/visible spectra. All the TD-DFT calculations were performed using the Gaussian 09 software package.

In order to further study the light-induced charge transfer (CT) properties of dye-sensitized molecules, the TD-DFT//BHandHLYP/6-311g(d,p) method was applied on the basis of the optimized structure of the ground state. And Multiwfn 3.6(dev) program^37,41^ was used for hole–electron analysis, such as the hole–electron distribution diagram, the charge density difference diagram, the centroid distance between the hole and the electron (*D* index), the average extension degree of the hole and the electron in CT direction (*u*_CT_ index), the overlap degree of the hole and the electron (*S*_m_ index), the binding energy of exciton (*E*_coul_) and the charge transfer amount from HOMO to LUMO orbital (*Q*_CT_) were calculated. The value of *Q*_CT_ was calculated through interfragment charge transfer (IFCT) method.^41^

In the process of electron excitation, the charge transfer from a fragment R of a molecule to a fragment S is defined as following:^41^

2 *Q*_R,S_ = *Θ*_R,hole_ × *Θ*_S,ele_

where *Θ*_R,hole_ is the hole percentage that fragment R accounts for in the process of light excitation. *Θ*_S,ele_ is the electron percentage that fragment S accounts for in the process of light excitation. 0 ≤ *Q*_R,S_ ≤ 1, and if the value of *Q*_R,S_ is larger, the R → S transfer will be more.

If the *D* index is larger, the hole and electron body will be distributed farther. We define the *D* index as:^41^

3 *D*_*x*_ = |*X*_ele_ − *X*_hole_|

4 *D*_*y*_ = |*Y*_ele_ − *Y*_hole_|

5 *D*_*z*_ = |*Z*_ele_ − *Z*_hole_|

6
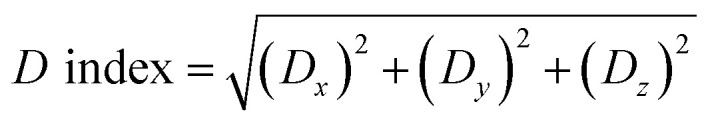
where *X*_hole_, *Y*_hole_, *Z*_hole_ respectively refers to the *X*, *Y*, *Z* coordinate of the hole centroid. *X*_ele_, *Y*_ele_, *Z*_ele_ respectively refers to the *X*, *Y*, *Z* coordinate of the electron centroid. *D*_*x*_, *D*_*y*_, *D*_*z*_ refers to the centroid distance between the hole and the electron in the *X*, *Y*, *Z* direction, respectively. The so-called centroid is equivalent to the most representative point of the overall functional distribution.

If the *H*_CT_ index is larger, the average extension of the hole and the electron in charge transfer direction will be larger. The *H*_CT_ index is defined as following:^41^

7 *H*_CT_ = |*Hu*_CT_|

8 *H* = (|*σ*_ele_| + |*σ*_hole_|)/2

where *u*_CT_ is the unit vector in charge transfer direction, *σ*_ele_, *σ*_hole_ refer to the distribution breadth index of the electron and the hole, respectively.

If the *S*_m_ index is larger, the overlap degree between the hole and electron will be larger. The *S*_m_ index is defined as following:^41^9
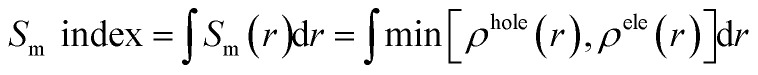
where *r* is the coordinate vector, *ρ*^hole^(*r*), *ρ*^ele^(*r*) is respectively the spatial distribution region of the hole and the electron.

Electron is negatively charged. And the place where the electron leaves, namely, the hole, which is positively charged accordingly. Therefore, there is coulomb attraction energy between the electron and the hole. This coulomb attraction energy, also known as exciton binding energy (*E*_coul_), can be calculated according to the following formula:^41^10
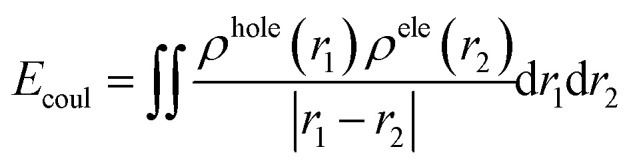
where *r* is the coordinate vector, *ρ*^hole^(*r*_1_), *ρ*^ele^(*r*_2_) is respectively the spatial distribution region of the hole and the electron. Smaller value of *E*_coul_ means weaker attraction between the hole and the electron and easier dissociation of the exciton.

All the electronic properties, optical properties and charge transfer properties of dyes D1–D4 were obtained based on the optimized geometries by DFT and TD-DFT theory.^42–49^

## 3. Results and discussion

### 3.1 Molecular structures and electronic property

**Fig. 2:**
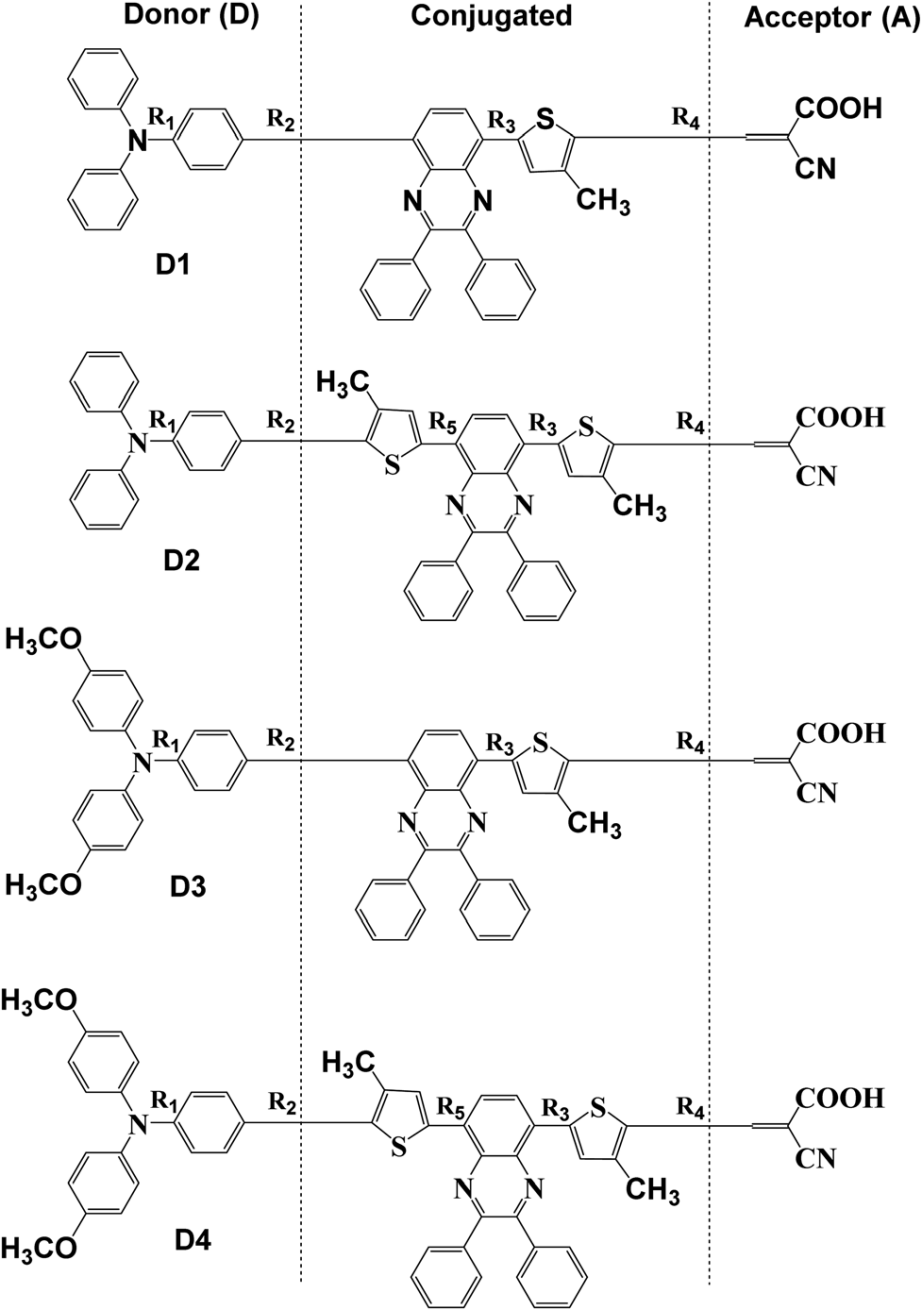
Molecular structures of dyes D1–D4.

The service life of dye-sensitized solar cells is closely related to the stability of the dyes. The stability of chemical molecules can be evaluated by the bond length and bond energy of chemical bonds.^42^ Shorter bond length means greater bond energy and more stable chemical molecules. More details about the ground-state optimized geometries of dyes D1–D4 are given in the ESI.†Table 1 shows the bond lengths *R*1–*R*5 denoted in Fig. 2 and single point energy (*E*) dyes D1–D4. Generally, the differences between the corresponding bond length data of dyes D1–D4 are not large. But when the triphenylamine group is connected with the electron-donating alkoxy group, the bond length of *R*1 decreases slightly. And when the triarylamine group is directly connected to the electron-withdrawing quinoline group, rather than the thiophene group, the bond length of *R*2 is slightly shorter than that of the latter. So we believe that the D1 molecule has the lowest stability, while the D4 molecule has the highest stability. What is more, the sequence of single point energy (*E*) is D4 < D2 < D3 < D1. Based on the analysis of bond lengths and single point energy above, it can be predicted that the stability of D4 is the highest and the stability of D1 is the lowest. Meantime, it can be obtained from the data of single point energy that the introduction of alkoxy group and thiophene group into triarylamine electron donor and conjugated bridge respectively, especially the introduction of thiophene, can reduce the energy of molecules, so as to improve the stability of molecules.

**Table 1:** The optimized distances by DFT//B3LYP/6-311G(d,p) and calculated single point energy by DFT//B3LYP/def2TZVP for dyes D1–D4

Dyes		*R*_1_/Å	*R*_2_/Å	*R*_3_/Å	*R*_4_/Å	*R*_5_/Å	*E*/kcal mol^−1^
In the vacuum	D1	1.413	1.477	1.462	1.422		−1.618 × 10^6^
	D2	1.415	1.468	1.460	1.429	1.459	−1.989 × 10^6^
	D3	1.408	1.475	1.462	1.419		−1.762 × 10^6^
	D4	1.410	1.467	1.458	1.420	1.458	−2.133 × 10^6^
In the solvent	D1	1.412	1.477	1.462	1.418		−1.618 × 10^6^
	D2	1.414	1.468	1.460	1.426	1.460	−1.989 × 10^6^
	D3	1.405	1.475	1.461	1.416		−1.762 × 10^6^
	D4	1.406	1.467	1.460	1.416	1.461	−2.133 × 10^6^

The planarity is important to dyes. Thus, in Fig. 1 and Table 2, all the dihedral angles between triphenylamine and thiophene, thiophene and quinoline have also been calculated by DFT//B3LYP/def2TZVP for dyes D1–D4. It is obvious that the introduction of thiophene group into conjugated bridge makes for increasing the dihedral angles between thiophene and quinoline, and thus decreasing the planarity of dye molecules, which may be favourable for reducing the π–π interchain interactions.^49^

**Table 2:** The dihedral angles (°) by DFT//B3LYP/def2TZVP for dyes D1–D4

Dyes	*θ*_1_/Å	*θ*_2_/Å	*θ*_3_/Å
D1	39.43	16.63	—
D2	38.00	18.09	11.03
D3	38.59	15.63	—
D4	37.05	22.00	16.08

The charge population of the electron donor, the conjugated bridge, as well as the electron acceptor of dyes D1–D4 is presented in Table 3 using Multiwfn 3.6(dev) program and NBO analysis. Here the effect of the introduction of alkoxy group and thiophene group on the molecular charge distribution was primarily investigated, so the π bridge of thiophene and the auxiliary acceptor of quinoline were regarded together as part of the conjugated bridge to be calculated, as shown in Fig. 2. The calculated results of charge population show that the charge number of the electron donor of triarylamine and the conjugated bridge is positive, while the charge number of the electron acceptor of cyanoacrylic is negative. This is because the four kinds of dyes D1–D4 all have the push–pull electronic structures, among which D1 and D3 are both possessed with the typical D–A′–π–A configurations, D2 and D4 are both possessed with the typical D–π–A′–π–A configurations. In addition, comparing D1, D2 with D3, D4 respectively, it is found that the introduction of alkoxy group significantly increased the positive charge number of the electron donor and the negative charge number of the electron acceptor. Comparing D1, D3 with D2, D4 respectively, it is found that the introduction of thiophene group also significantly increased the positive charge number of the electron donor and the negative charge number of the electron acceptor. What has been compared above indicates that the introduction of alkoxy group and thiophene group makes for enhancing the electron-donating ability of the triarylamine donor and the intramolecular charge transfer from the triarylamine donor to the cyanoacrylic acid acceptor.

**Table 3:** Charge population of dyes D1–D4 by DFT//B3LYP/6-311G(d,p)

Dyes	Donor (D)	Conjugated	Acceptor (A)
D1	0.054*e*	0.078*e*	−0.132*e*
D2	0.027*e*	0.156*e*	−0.183*e*
D3	0.071*e*	0.064*e*	−0.135*e*
D4	0.042*e*	0.186*e*	−0.227*e*

**Fig. 3:**
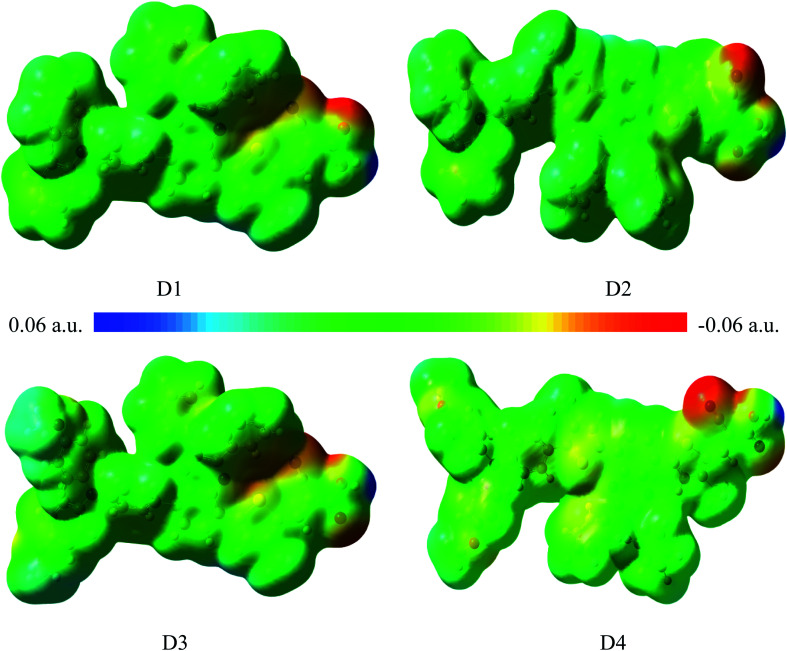
Electrostatic potential distribution (ESP) of dyes D1–D4 by DFT//B3LYP/6-311G(d,p).

Fig. 3 shows the surface electrostatic potential (ESP) distribution of the dyes D1–D4 obtained by DFT//B3LYP/6-311g(d,p). In the ESP distribution diagram, the color red corresponds to the negative ESP area, while the color blue corresponds to the positive ESP area. The distribution of higher positive value indicates there exists stronger negative charge, and the distribution of higher negative value indicates there exists stronger positive charge.^43,44^Fig. 3 shows the blue regions are mainly distributed on the electron donor of triarylamine and conjugated bridge, and the red regions are mainly distributed on the electron acceptor of cyanoacrylic acid, which is consistent with the analysis of charge population. It is shown that the electronic push–pull structures of the dyes are beneficial to the charge separation. In order to further study their charge separation degree, the dipole moments^45^ of the dyes D1–D4 are listed in Table 4. The calculated results suggest that the order of the dipole moments is D4 > D3 > D2 > D1, that is, the charge separation degree of D4 is the largest, and the charge separation degree of D1 is the smallest. This is due to the reason that compared with D1, the introduction of alkoxyl group and thiophene group of D4 facilitates the transfer of electrons from the donor to the acceptor.

**Table 4:** Dipole moments of dyes D1–D4 by DFT//B3LYP/6-311G(d,p)

Dyes	Dipole moment/D
D1	7.02
D2	7.39
D3	8.17
D4	10.04

### 3.2 The frontier molecular orbitals

It is well known that the highest occupied molecular orbital (HOMO) and lowest unoccupied molecular orbital (LUMO) of dyes are of great significance, because the frontier orbital energy levels and energy gaps of molecules are closely related to the molecular excitation and transition properties, and are the key factors to determine the photoelectric conversion efficiency (PCE) of solar cells.^24–26,29^

**Fig. 4:**
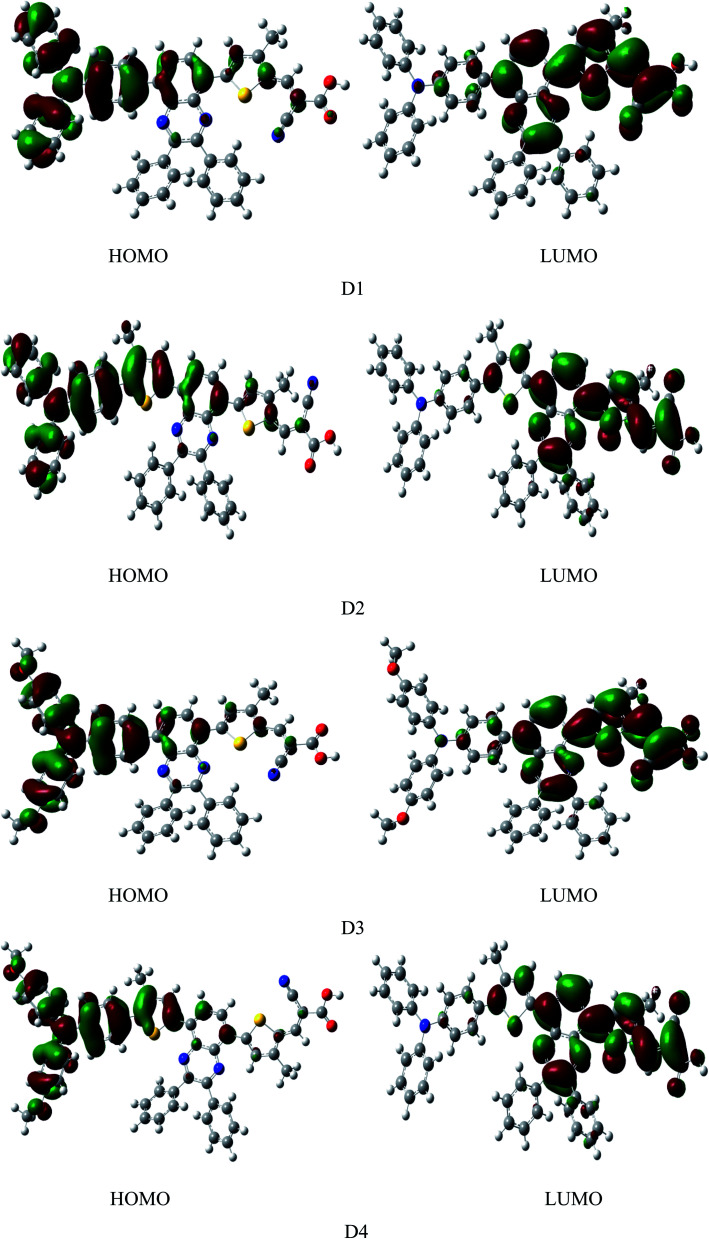
The frontier molecular orbitals of dyes D1–D4 by DFT//B3LYP/6-311G(d,p) in vacuum.

Fig. 4 shows the frontier molecular orbital diagram of dyes D1–D4 simulated by DFT//B3LYP/6-311g(d,p) in vacuum. The diagram shows that the HOMOs of dyes are mainly distributed on the electron donor as well as the conjugated bridge attached to donor, LUMOs of dyes are mainly distributed from electron acceptor of the cyanoacrylic acid to the quinoline group. On the one hand, HOMO and LUMO have some overlaps on the conjugated bridge, which is the prerequisite of intramolecular charge transfer.^46^ On the other hand, HOMO and LUMO can be well-separated, which contributes to the light-induced intramolecular charge transfer from donor to acceptor.^47^ HOMOs, containing ground state characteristics,^48^ are mostly the π orbitals of the electron donor and conjugated bridge attached to donor where the ground-state electrons of dyes are mainly located. LUMOs, containing excited state characteristics,^48^ are mostly the single state π* orbitals distributed from the electron acceptor cyanoacrylic acid to the quinoline group where the excited-state electrons of dyes are mainly located. Under illumination conditions, the dye molecules, with the anchor group of cyanoacrylic acid absorbed on the surface of semiconductor, transit from the ground state to the excited state by π → π* transition. This process happens inevitably accompanied by the electrons transferring from donor to acceptor through intramolecular charge transfer^49^ and then injected into the semiconductor thin film.

**Fig. 5:**
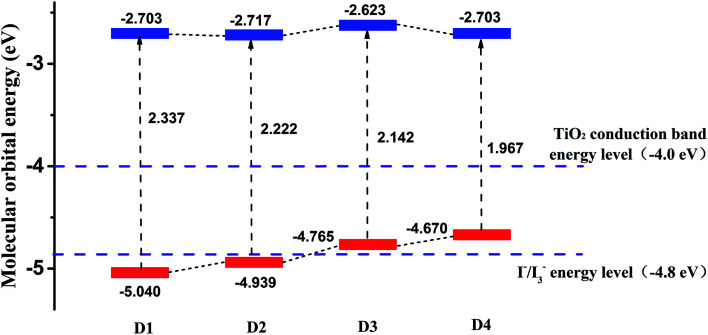
The frontier molecular orbital energy levels of dyes D1–D4 by DFT//B3LYP/6-311G(d,p).

Fig. 5 shows the calculated frontier molecular orbital energy levels and energy gaps (Δ*E*_H–L_) of dyes D1–D4 by DFT//B3LYP/6-311g(d,p). As is shown in Fig. 5, the lowest unoccupied orbital (LUMO) energy levels of the dyes D1–D4 are all much higher than TiO_2_ conduction band level (−4.0 eV),^50^ which is conducive to the smooth injection of electrons into the semiconductor film (TiO_2_) after the light excitation of the dyes. And the highest occupied orbital (HOMO) energy levels of the dyes D1–D4 are all near the I^−^/I_3_^−^energy level (−4.8 eV). The highest occupied orbital (HOMO) energy levels of the dyes D3 and D4 are slightly higher than the I^−^/I_3_^−^ energy level (−4.8 eV). On the one hand, considering the calculation error and all the calculations were simulated in vacuum condition, it can be acceptable for the theoretical calculation. On the other hand, the LUMO levels of dyes D3 and D4 are preeminently above the conduction band of TiO_2_ (4.0 eV), indicating that the electron injection from the excited dyes to the conduction band of the TiO_2_ can be capable and feasible, while the HOMO levels the dyes D3 and D4 are slightly higher than the I^−^/I_3_^−^ energy level (−4.8 eV).^49,51^ Hence it can be seen that all the dyes follow the energy level matching principle. Generally, when the LUMO energy level of dyes is higher, the driving force of dyes to inject electrons into the TiO_2_ will be greater,^48^ which is beneficial for higher PCE of solar cells. The differences between the LUMO energy level of dyes D1–D4 and the TiO_2_ conduction band level are 1.3 eV, 1.3 eV, 1.4 eV, 1.3 eV, respectively. These differences are very small, so it can be concluded that the abilities of dyes D1–D4 injecting electrons into TiO_2_ thin film are almost the same. In addition, the Fig. 5 also reflects that the introduction of alkoxyl chains and thiophene group significantly contributes to improving the HOMO energy level of dyes, but has little effect on the LUMO energy level.

As is known that the molecular energy gap (Δ*E*_H–L_) has an important effect on the electron absorption spectra. And narrow energy gap is more favorable for extending the wavelength range of solar cells, which can be seen from Fig. 5. The energy gaps of dyes D1–D4 are 2.337 eV, 2.222 eV, 2.142 eV, 1.967 eV respectively. And the order of the energy gaps is D4 < D3 < D2 < D1. This is because the introduction of alkoxyl chains and thiophene group is conducive to improving the HOMO energy level and reducing the energy gap.^52–54^ Therefore, it can be predicted that the absorption band of dye D4 in the visible region is more advantageous than the other three dyes D1–D3. In order to further study the optical properties of the four dyes, then the electron spectra of dyes D1–D4 were simulated.

### 3.3 Absorption spectra

**Fig. 6:**
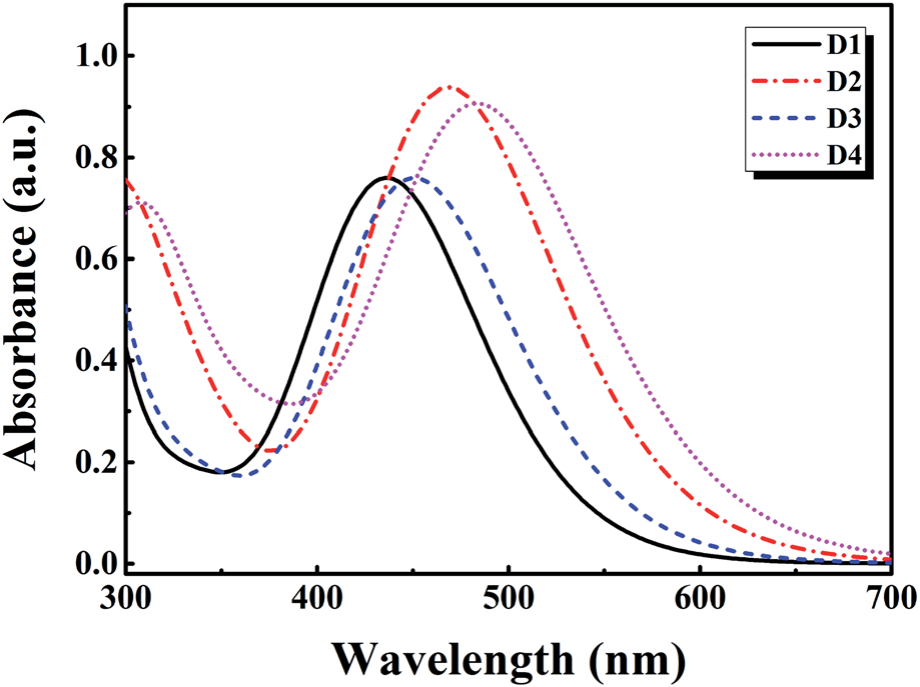
The UV-VIS absorption spectra of dyes D1–D4 by TD-DFT//BHandHLYP/6-311G(d,p).

Fig. 6 shows the absorption spectra of dyes D1–D4 simulated by TD-DFT//BHandHLYP/6-311g(d,p) in vacuum. Table 5 shows the absorption spectra properties, including maximum absorption wavelength (*λ*_max_), excitation energy (*E*_ex_), oscillator strength (*f*), light harvesting efficiency (LHE) and major contributions of the calculated transitions. As is displayed in Table 5 and Fig. 6, it can be found that all the dyes have strong absorption bands between 400 nm and 700 nm, and their maximum absorption peaks are mainly from the HOMO → LUMO transition, which can be attributed to intramolecular charge transfer.^49^ The oscillator strength at the maximum absorption peak of dyes D1–D4 is 1.12, 1.38, 1.11 and 1.35 respectively. And the corresponding LHE of dyes D1–D4 is 0.92, 0.96, 0.92 and 0.96 respectively. This indicates that the introduction of thiophene group into conjugated bridge can significantly improve the light harvesting efficiency of dyes, while the introduction of alkoxy group into the triarylamine donor has little effect on the light harvesting efficiency of dyes. The maximum absorption wavelength of dyes D1–D4 is 437 nm, 469 nm, 448 nm, 485 nm, respectively. And the corresponding excitation energy of dyes D1–D4 is 2.84 eV, 2.64 eV, 2.77 eV, 2.56 eV, respectively. The order of the maximum absorption wavelength is D4 > D2 > D3 > D1, and the order of excitation energy is D4 < D2 < D3 < D1. Obviously, compared with D1 and D3, the maximum absorption peaks of D2 and D4 are significantly red-shifted. This is because the introduction of thiophene group assists in enhancing the conjugated degree of dyes, thus strengthening the intramolecular charge transfer, and causing the red-shift of absorption spectra. Besides, compared with D2, the maximum absorption wavelength of D4 has a slight red-shift. This is due to the introduction of alkoxy groups. In addition, it can be shown that the maximum absorption wavelength at the visible region corresponds to the S0 → S1 transition. These results are consistent with relevant ref. 52–54. Based on the above analysis, we believe that D2 and D4 have better optical properties than D1 and D3, among which D4 has the optimal optical properties.

**Table 5:** The absorption spectral properties of dyes D1–D4 by TD-DFT//BHandHLYP/6-311G(d,p)

Dyes	Electronic transitions	*λ*_max_/nm	*E*_ex_/eV	*f*	LHE	Main configurations
D1	S0 → S1	437	2.84	1.12	0.92	HOMO → LUMO (67%)
D2	S0 → S1	469	2.64	1.38	0.96	HOMO → LUMO (72%)
D3	S0 → S1	448	2.77	1.11	0.92	HOMO → LUMO (72%)
D4	S0 → S1	485	2.56	1.35	0.96	HOMO → LUMO (65%)

### 3.4 Charge transfer properties

As is known, the dyes can be stimulated when exposed to light, and the electrons jump from the HOMO orbital (ground state) to the LUMO orbital (excited state). In this way, the electron–hole pairs (excitons) are generated, and the excitons will be dissociated to form free charges, which is the first process that occurs in the photoelectric conversion process of organic solar cells.^55–57^ Through the Multiwfn 3.6(dev) program, the spatial distribution of the holes and electrons, the charge transfer amount of HOMO → LUMO (*Q*_CT_), the centroid distance between the hole and the electron (*D* index), the average extension degree of the hole and the electron in CT direction (*u*_CT_ index), the overlap degree of the hole and the electron (*S*_m_ index), and the binding energy of exciton (*E*_coul_) can be obtained to investigate the charge transfer properties of dyes. The study object is the first excited state of dyes, because the first excited state corresponds to the maximum absorption wavelength according to the spectral analysis.

**Fig. 7:**
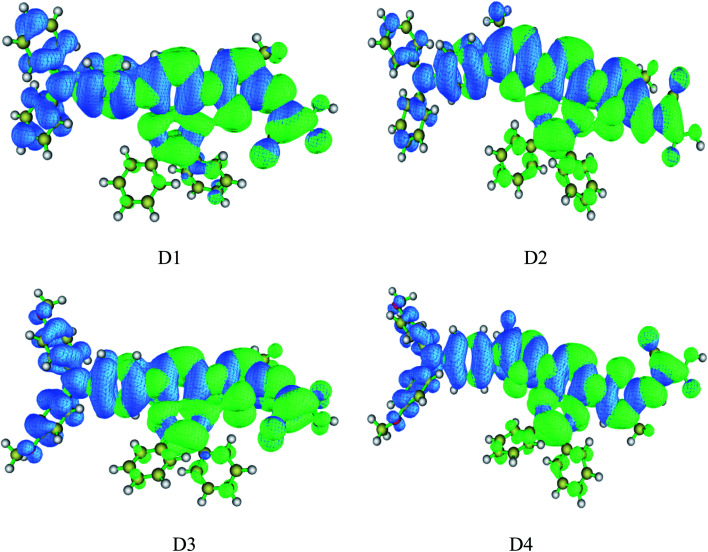
Hole–electron distribution map of dyes D1–D4 by TD-DFT//BHandHLYP/6-311G(d,p) (blue represents hole distribution, electron represents electron distribution).

Hole distribution is to describe where the stimulated electrons come from, and the electron distribution is to describe where the stimulated electrons go. The Fig. 7 shows the hole–electron distribution map, and the color blue represents for the hole distribution, the color green for electron distribution. It is shown that the distribution of hole (blue area) is mainly on the electron donor as well as the conjugated bridge attached to it, and the distribution of electron (green area) is mainly from electron acceptor of the cyanoacrylic acid to the quinoline group, which indicates the charge transfer from donor to acceptor in the process of photoexcitation, consistent with previous charge population analysis and HOMO–LUMO orbital analysis.

According to the previous frontier orbital analysis, the HOMO orbitals are mainly distributed on the arylamine donor and delocalized to the conjugated bridge, and the LUMO orbitals are mainly distributed on the cyanoacrylic acid acceptor and delocalized to the quinoline group. So the distribution fragments of HOMO and LUMO were selected to compute the charge transfer amount (*Q*_CT_) from HOMO to LUMO. The reason to inquire into the charge transfer of HOMO → LUMO is that the HOMO → LUMO transition corresponds to the intramolecular charge transfer and the maximum absorption. Because of the involvement in photoexcitation process, the calculated functional and basis set of *Q*_CT_ are consistent with the calculated method for absorption spectra. Table 6 shows the calculated results of HOMO → LUMO charge transfer amount (*Q*_CT_). It is signified that the order of *Q*_CT_ is: D4 > D3 > D2 > D1, and more *Q*_CT_ is conducive to greater charge transfer as well as higher photoelectric conversion. Furthermore, it is also shown that the introduction of alkoxy groups and thiophene group can enhance the charge transfer from HOMO to LUMO, which is consistent with the previous absorption spectra analysis.

**Table 6:** The calculated photoinduced charge transfer amount of dyes D1–D4 by TD-DFT//BHandHLYP/6-311G(d,p)

Dyes	D1	D2	D3	D4
*Q*_CT_	0.56*e*	0.60*e*	0.63*e*	0.68*e*

On the other hand, efficient separation of excitons is beneficial for better charge transfer and photoelectric conversion. Thus the *D* index, *u*_CT_ index, *S*_m_ index and *E*_coul_ were calculated to further investigate the hole–electron distribution and the dissociation efficiency of electron–hole pairs (excitons). Table 7 shows the calculated results by TD-DFT//BHandHLYP/6-311g(d,p). It is shown that the *D* index of dyes D1–D4 is 2.78 Å, 2.67 Å, 3.79 Å, and 3.77 Å respectively, which indicates that the introduction of alkoxyl groups can significantly improve the *D* index of the molecule, while the introduction of thiophene group can slightly but not significantly reduce the *D* index. The *H*CT index of dyes D1–D4 is 4.43 Å, 4.49 Å, 4.53 Å, and 4.75 Å respectively, indicating that the introduction of alkoxyl groups and thiophene group could improve the molecular *H*_CT_ index. The *S*_m_ index of dyes D1–D4 is 0.46 Å, 4.46 Å, 4.41 Å, and 4.42 Å respectively, indicating that the introduction of alkoxy groups could reduce the *S*_m_ index, while the introduction of thiophene group has almost no effect on the *S*m index. Through the analysis of *D* index, *H*_CT_ index and *S*_m_ index, it can be seen that the introduction of the alkoxy on the one hand can improve the *H*_CT_ index of molecules so as to increase the average extension degree and delocalization degree of the hole and electron distribution in the direction of charge transfer, which is favorable for hole and electron transfer, on the other hand can improve the *D* index and reduce the *S*_m_ index, so as to make the hole distribution far away from the electron distribution and reduce the overlap of hole and electron distribution, which is advantageous to the dissociation of excitons. The introduction of thiophene group on the one hand can improve the *H*_CT_ index of molecules to strength the hole and electron transfer, on the other hand can slightly reduce the molecular *D* index, which is also reflected in the hole–electron distribution map and charge density difference map.

**Table 7:** The calculated *D* index, *H*_CT_ index, *S*_m_ index and excition binding energy (*E*_coul_) for dyes D1–D4 by TD-DFT//BHandHLYP/6-311G(d,p)

Dyes	*D* index/Å	*H*_CT_/Å	*S*_m_ index/Å	*E*_coul_/eV
D1	2.78	4.43	0.46	3.49
D2	2.67	4.49	0.46	3.41
D3	3.79	4.53	0.41	3.26
D4	3.77	4.75	0.42	3.15

The exciton binding energy (*E*_coul_) of dyes D1–D4 is 3.49 eV, 3.41 eV, 3.26 eV and 3.15 eV respectively, and the order of *E*_coul_ is: D4 < D3 < D2 < D1. It is clearly seen that D3 and D4 have higher exciton dissociation efficiency than D1 and D2, and the exciton dissociation efficiency of D4 is the highest, indicating that the introduction of alkoxy groups and thiophene group can reduce the exciton binding energy of dyes and improve the exciton dissociation efficiency, which is advantageous to the charge transfer.

## 4. Conclusion

We have systematically studied the electronic properties, optical properties and charge transfer properties of dyes D1–D4 by DFT and TD-DFT theory. The calculated results of bond length and single point energy indicates that the chemical stability of dyes D1–D4 follow this sequence: D4 > D3 > D2 > D1. The simulated results of charge population, ESP distribution and dipole moments show that the introduction of alkoxy groups into triarylamine donor and thiophene group into conjugated bridge can promote the charge separation of dyes, and the degree of charge separation is in order: D4 > D3 > D2 > D1. The calculated absorption spectra properties, including maximum absorption wavelength, excitation energy, oscillator strength and light harvesting efficiency, reveal that the introduction of thiophene group into the conjugated bridge can significantly improve the light harvesting efficiency, reduce the excitation energy and cause obvious red-shift, thus D2 and D4 show better optical properties than D1 and D3, besides, D4 has the optimal optical properties. The results of hole–electron analysis display that the order of charge transfer amount from HOMO to LUMO (*Q*_CT_) is: D4 > D3 > D2 > D1. In addition, the introduction of alkoxy groups can improve the *H*_CT_ index, *D* index and reduce the *S*_m_ index of dyes. And the introduction of thiophene group can improve the *H*_CT_ index and slightly reduce the *D* index. What is more, the exciton binding energy (*E*_coul_) of dyes D1–D4 is in order: D4 < D3 < D2 < D1. And smaller binding energy makes for higher separation efficiency of excitons and better charge transfer.

Based on the above analysis, it can be concluded that D4 has more advantages in molecular properties, optical properties and charge transfer properties than the other three dyes (D1, D2 and D3). And these advantages are conducive to the photoelectric conversion. We believe that dye D4, which has been introduced in alkoxy groups as well as thiophene group and possesses D–π–A′–π–A configuration, is a more ideal dye sensitizer.

## Conflicts of interest

There are no conflicts to declare.

## Supplementary Material

RA-010-D0RA01040E-s001
