# Supplementary material for: Theoretical study of D–A′–π–A/D–π–A′–π–A triphenylamine and quinoline derivatives as sensitizers for dye-sensitized solar cells
Source: RSC Adv. 2020 May 4;10(29):17255–65. doi: 10.1039/d0ra01040e (PMC9053390; doi:10.1039/d0ra01040e)
Supplement: RA-010-D0RA01040E-s001 [file RA-010-D0RA01040E-s001.pdf]

Electronic Supplementary Material (ESI) for RSC Advances.
This journal is © The Royal Society of Chemistry 2020

Supporting information for:

Theoretical study of D-A'-π-A / D-π-A'-π-A triphenylamine and quinoline derivatives as sensitizer for dye-sensitized solar cells

Ying Zhang,[‡a] Ji Cheng,[‡a] Wang Deng,[‡b] Bin Sun,[c] Zhixin Liu,[a] Lei Yan, *[a] Xueye Wang, *[a] Baoming Xu,*[b] Xingzhu Wang *[a,b]

[a] *College of Chemistry, School of Physics and Optoelectronics, Xiangtan Univeristy, Xiangtan, Hunan Province 411105, China*

[b] *Department of Materials Science & Engineering and Academy for Advanced Interdisciplinary Research, Southern University of Science and Technology, Shenzhen, Guangdong Province 518055, China,*

[c] *North China Sea Marine Forecasting Center of Ministry of Natural Resources, Qingdao, Shandong Province 266000, China*

**Corresponding authors.*

E-mail addresses: xz_wang@hotmail.com (X.Z. Wang); yanlei@xtu.edu.cn (L. Yan);wxueye@xtu.edu.cn (X. Wang); xubm@sustech.edu.cn(B.Xu)

‡ Those authors contributed equally.

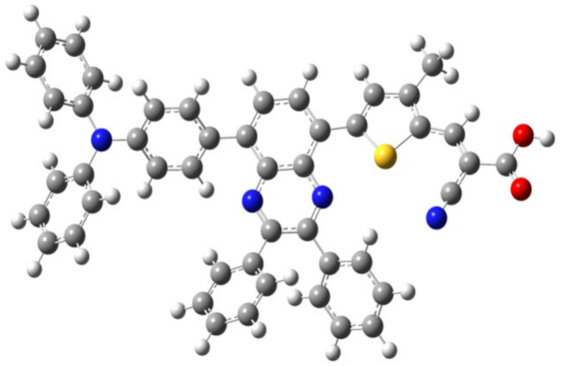

D1

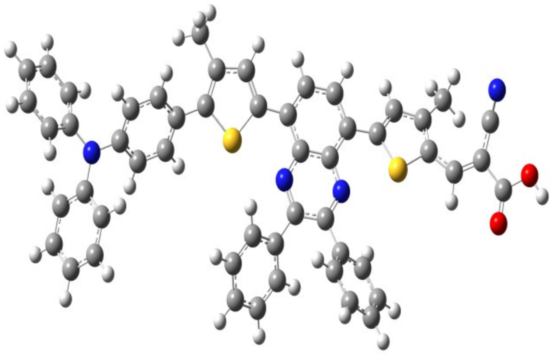

D2

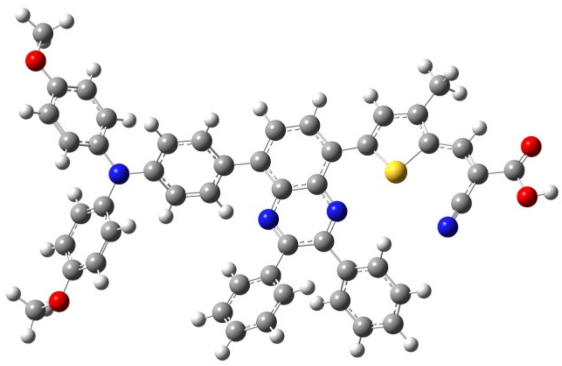

D3

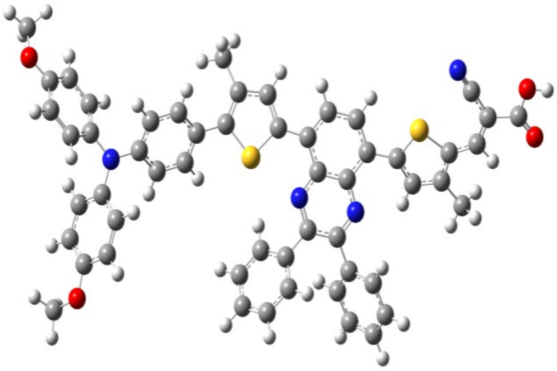

D4

Figure S1. The optimized structures of dyes D1-D4 by DFT//B3LYP/6-311G(d,p)
